# Parental alcohol intoxication and adverse health outcomes among offspring. A 4-year follow up HUNT study among 2399 Norwegian adolescents

**DOI:** 10.1016/j.pmedr.2020.101170

**Published:** 2020-08-04

**Authors:** S.H. Haugland, L. Coombes, A. Strandheim

**Affiliations:** a Faculty of Health and Sport Sciences, Department of Psychosocial Health, University of Agder, Postboks 422, 4604 Kristiansand, Norway; b Faculty of Life and Health Sciences, Department of Psychology, Social Work and Public Health, Oxford Brookes University, Gypsy Lane, Headington, Oxford OX3 0BP, United Kingdom; c Regional Centre for Child and Youth Mental Health and Child Welfare, Norwegian University of Science and Technology, Norway; d The Department of Child and Adolescent Psychiatry, Nord-Trøndelag Health Trust, 7600 Levanger, Norway

**Keywords:** Alcohol drinking, Alcohol intoxication, Parents, Adolescent health

## Abstract

This study aimed to investigate whether experience of parental alcohol intoxication was associated with adverse health outcomes among adolescents four years later. A population-based Norwegian cohort study of 2399 adolescents who participated in the Young-HUNT1 Survey 1995–97 (T1, 13–15 years old) was followed up four years later (T2) in 2000 (Young-HUNT2, 17–19 years old). Measures were based on adolescent self-report of exposure to parental alcohol intoxication, self-rated general health, mental distress (SCL-5) and lifetime hospital admission. Multivariable logistic regression analyses, adjusted for gender, age and parental education were applied. Results show that half of the adolescents (51%) had seen parents intoxicated at age 13–15 years. Four years later, those who had reported parental alcohol intoxication at T1 had increased odds of admission to hospital with odds ratios ranging from OR1.3; CI 1.0–1.7 to OR 2.2; CI 1.3–3.9, poorer self-rated health (odds ratio ranging from 1.8;1.2–2.6 to 2.0;1.1–3.7) and more mental distress (odds ratio ranging from 1.7;1.1–2.5 to 1.9;1.0–3.6). Furthermore, the increased frequency of experience of parental alcohol intoxication are associated with higher prevalence of admission to hospital and mental distress and lower levels of self-rated health. Findings from this large, representative population of Norwegian adolescents indicate that adverse health outcomes among adolescents in a general population are related to relatively common heavy drinking behaviours among parents.

## Highlights

• Alcohol’s harm to others includes adverse health outcomes in adolescence.
• Non-dependent parental heavy drinking increases health harm among adolescents.
• Hospital admission was related to exposure to parental alcohol intoxication.
• Mental distress was related to exposure to parental alcohol intoxication.
• Poor self-rated health was related to exposure to parental alcohol intoxication.

## 1 Introduction

Alcohol use may cause substantial health loss. It was the seventh leading risk factor for death and disability (DALY) globally in 2016 (Griswold, 2018), and the eighth in Norway in 2017 (IHME, 2020). Most of the literature on alcohol related harm focuses on the consequences for the alcohol user, and more prospective studies are needed to assess the harm to others caused by the alcohol use of someone in their environment (Griswold, 2018). A body of research on *alcohol's harm to others* (AHTO) is emerging globally to explore the range of second-hand effects of alcohol use (Karriker-Jaffe, 2018, Warpenius and Tigerstedt, 2016). The World Health Organization (WHO) focuses on AHTO in its Global strategy to reduce the harmful use of alcohol (WHO, 2010).

Family plays the most prominent role in upbringing worldwide during childhood and adolescence (Bailey, 2009, Salom, 2014). Young people may be especially vulnerable to harm from alcohol use from a parent as they have little control over their situation. Research estimates regarding the proportion of children living with alcohol-misusing parents vary between countries (EMCDDA, 2010, Dawe, 2007, Manning et al., 2009) and living with heavy drinkers has been found to increase the risk of harm (Casswell et al., 2011, Laslett, 2017). Moreover, there is some evidence that children with parents diagnosed with alcohol disorders experience adverse health outcomes, both physically and mentally (Christensen and Bilenberg, 2000, Johnson and Leff, 1999, Christoffersen and Soothill, 2003, Hussong, 2008, Hussong, 2010, Jaaskelainen, 2016). Children within such a context are also at higher risk for injuries and accidents that may lead to hospitalisation (Raitasalo, 2015, Orton, 2012, Baker, 2015, Winqvist, 2007).

Although the number of children living with alcohol misuse/dependence is significant, a far larger number of children live with alcohol-using parents whose use does not meet the diagnostic criteria for alcohol disorders. However, there has been considerably less examination of the potential harm to children's health resulting from occasional parental heavy alcohol use (Rossow, 2016, McGovern, 2018). Some studies have found that adverse health outcomes are related to parental non-dependent alcohol use (McGovern, 2018), but the evidence base is weak and findings are inconclusive (Raitasalo, 2015, Rossow, 2016, McGovern, 2018, Mahedy, 2017, Malone et al., 2010). Most of the alcohol-related harm among alcohol users is not caused by alcohol disorders but is a result of more common hazardous drinking patterns, such as heavy episodic drinking (Danielsson, 2012, Skog, 1999). From a public health perspective, it is crucial to investigate whether such drinking patterns also increase the risk of harm to people other than the drinker (Karriker-Jaffe, 2018). Especially pertinent is the investigation of harm drinkers cause their children, as children are vulnerable and dependent on adults. Drinking to intoxication and being drunk are also phenomena far more prevalent than alcohol misuse and represent a relatively common drinking pattern among Norwegians, including parents (Haugland, 2013, Haugland et al., 2015). Heavy drinking episodes are also quite common in Europe (Ferreira-Borges, et al., 2019). Therefore, investigating whether exposure to parental alcohol intoxication is associated with adverse health outcomes among adolescents would contribute new knowledge to the AHTO field.

The purpose of this study is to investigate whether self-reported exposure to parental alcohol intoxication in early adolescence was associated with adverse health outcomes among adolescents four years later.

Specifically, we would like to address the following research questions:

Is exposure to parental alcohol intoxication reported in early adolescence associated with self-reported hospital admission four years later?
Is exposure to parental alcohol intoxication reported in early adolescence associated with self-reported mental distress four years later?
Is exposure to parental alcohol intoxication reported in early adolescence associated with self-reported health four years later?

## 2 Materials and methods

### 2.1 Design and participants

The Nord-Trøndelag Health Study (HUNT) is the most extensive collection of population data in Norway, with data from 123000^1^ unique participants collected in several waves. The Nord-Trøndelag Health Study (The HUNT Study) is a collaboration between HUNT Research Centre (Faculty of Medicine and Health Sciences, Norwegian University of Science and Technology NTNU), Nord-Trøndelag County Council, Central Norway Regional Health Authority, and the Norwegian Institute of Public Health. For more information on the HUNT-Study, see Krokstad (2012).

As part of the HUNT-study, the Young-HUNT1 Survey was established in 1995–97. All adolescents aged 13–19 in the county were invited to participate, and 88.1% completed the questionnaire (n = 8983). All county school principals gave written consent to their school's participation, and lists of students formed the basis for the invitations. Students completed a questionnaire during school hour, and adolescents who were not registered as students were invited by post. The youngest participants (aged 13–16) constitute the baseline (T1) for a follow-up study four years later in Young-HUNT2 (T2) where 76% of them were eligible for invitation at T2. Those who were not invited at T2 were either attending the first year of high school, changed school courses, attended schools in other municipalities outside the county, quit school or moved out of the county. A total of 3124 participants at T1 was invited to take part at T2 and a total of 2399 adolescents participated in both surveys (Holmen, 2013). The use of personal id-numbers from the National Registry connected data from both surveys and facilitated longitudinal data at the individual level.

For more detailed information, see Holmen (2013).

### 2.2 Measures

All measures were based on adolescent self-reports.

#### 2.2.1 Explanatory variable

Parental alcohol intoxication was measured by asking respondents at baseline (T1) “Have you ever seen either of your parents intoxicated?”, with response categories: 1. Never; 2. A few times ever; 3. A few times a year; 4. A few times a month; 5. A few times a week. The latter two response categories were recoded to 'a few times a month or more often'. ‘Never’ was used as the reference category in multivariable analyses.

#### 2.2.2 Outcome variables

Information regarding hospital admission was retrieved by asking participants at T2 “Have you ever been admitted to the hospital (exclude when you were born)?”, and response categories were “No, never”, “Yes, once” and “yes, more than once”. The two latter categories were combined to create a variable measuring “hospital admission, ever”.

The participants reported their self-rated health (SRH) at T2 by answering the question “How is your health now”, with response categories bad, not quite good, good, very good. The variable was dichotomised, and categories 1&2 were defined as poor self-rated health.

A short version of the question battery of the Hopkins Symptom Checklist (SCL-5) was applied to measure mental distress at T2 (which has been found to perform almost as well as full version (Strand, 2003). Respondents reported whether over the last 14 days they had felt sad or depressed; felt hopeless about the future; felt tense or keyed up; constantly felt fearful and anxious; or worried a lot about everything. Response categories were ‘not bothered’, a little bothered’, ‘bothered quite a lot’ and ‘extremely bothered’. We totalled the responses and constructed a dichotomous variable in which a score of ≥2 was defined as indicating mental distress.

#### 2.2.3 Control variables

Parental education, adolescent age and gender were considered as possible confounders.

Parental education is relevant as studies indicate that parents with a higher level of education have access to and apply information relevant to parenting practices more rapidly than parents with lower education (Radey and Randolph, 2009). In addition, higher socio-economic status is associated with more alcohol use (Strand and Steiro, 2003). Exposure to parental intoxication also increase by age within similar populations (Haugland et al., 2015), and adolescent outcomes may vary by gender (Haugland, 2013).

Information on parental education variable was based on the pupils’ reporting at T2 of the parent with highest completed education. The measure does not identify which parent this refers to. The parents’ education was grouped into five categories: 1. Primary and lower secondary school; 2. Upper secondary school with vocational subjects, high school; 3. Upper secondary school with general subjects, high school; 4. University college/university education of less than four years’ duration; 5. University college/university education of four years or more. Categories 1–3 were defined as low education (reference category), and categories 4–5 were defined as high.

Age was applied as a continuous variable based on age at follow-up/T2 (Young-HUNT 2). Age was computed as the number of days between birth (as registered by the Norwegian National Registry) and the date in question, divided by 365.2425 (the average number of days per year in the Gregorian calendar), rounded to one decimal.

Adolescent gender was retrieved by asking respondents whether they were male or female at T2 (reference category).

### 2.3 Statistical analysis

Descriptive analyses were performed to provide an overview of the data, including frequencies, and 2×2 cross-tables with chi-square tests.

Multivariable logistic regression analyses were applied to explore possible associations between parental alcohol intoxication reported at baseline and self-rated health, mental distress and hospital admission four years later (adjusted for parental education, adolescent age and gender).

Results were reported by odds ratios (ORs), 95% confidence intervals (CIs) with p-values with a significance level set to 0.05 (P). Multivariable analyses were adjusted for parental education, age and gender.

Multivariable analyses exploring possible interactions between gender and parental alcohol intoxication were performed, as previous studies have shown that adolescent outcomes related to parental intoxication vary with gender (Haugland, 2013, Haugland et al., 2015).

### 2.4 Assessment of ethical considerations in the research

All participants, along with the guardians of participants under the age of 16, gave their written consent to take part in the study. The HUNT study, including Young-HUNT, was approved by the Norwegian Data Inspectorate and the Regional Committee for Medical Research Ethics (REK), and this current study has also been approved by REK (REK case number 2015/2090).

## 3 Results

### 3.1 Descriptive analyses

Table 1 provides a descriptive overview of variables applied in multivariable analyses. Over half (51.2%) of the adolescents reported having seen their parents intoxicated, but only 4% had seen them intoxicated monthly or more often. Adolescents’ mean age at follow-up (Young-HUNT2) was 18.4 years.

**Table 1:** Descriptive statistics of exposure variables, outcome variables and control variables. The adolescent part of the Nord-Trøndelag Health Survey (Young-HUNT 1 &2) 1995–97 and 2000.

Variables		% (n)	Mean (SD) (n)
Exposure variables (T1)	Never seen parents intoxicated	48.9 (1120)	
	Seen parents intoxicated a few times ever*	32.2 (739)	
	Seen parents intoxicated a few times a year	14.9 (341)	
	Seen parents intoxicated monthly or more often	4.0 (92)	
Dependent variables (T2)	Self-rated health, poor	12.2 (291)	
	Mental distress	10.8 (256)	
	Hospital admission	42.5 (766)	
Control/background variables (T2)	Gender, boys	46.5 (1115)	
	Gender, girls	53.5 (1284)	
	Parental education, high	38.9 (825)	
	Age		18.4 (0.80) (2399)

* Lifetime.

Table 2 reveals twice as many reports of poor health among those who had seen parents intoxicated a few times a month or more often. Mental distress was equally prevalent among those who had seen parents intoxicated a few times a year and among those exposed to parental intoxication a few times a month or more often, but less common among those who never had seen parents alcohol intoxicated. All outcome-measures were more common among girls than boys. Poor self-rated health was slightly more common among adolescents with parents with lower education (13% vs 10%, p = .037), but mental distress and hospital admission did not differ according to parental education.

**Table 2:** Frequency of adverse health outcomes (T2) related to the exposure variable (T1) and control variables (T2) percentage, and chi-square test (<0.05). The adolescent part of the Nord-Trøndelag Health Survey (Young-HUNT1 &2) 1995–97 and 2000.

		Self-rated health, poor		Mental distress		Hospital admission	
		n (%)	p-value	n (%)	p-value	n (%)	p-value
Seen parents intoxicated	Never	112 (10.1)	p = .000	101 (9.1)	p = .008	326 (38.3)	p = .000
	A few times ever	83 (11.3)		81 (11.0)		245 (44.0)	
	A few times a year	61 (17.9)		51 (15.0)		122 (47.1)	
	A few times a month or more often	18 (20.0)		14 (15.2)		33 (55.0)	

Gender	Girls	178 (14.0)	P = .005	178 (14.0)	p = .000	405 (40.4)	p = 0.05
	Boys	113 (10.2)		78 (7.1)		361 (45.1)	
Parental education	Low	170 (13.1)	p = .037	135 (10.5)	*n.s*	406 (41.1)	*n.s*
	High	83 (10.1)		92 (11.2)		284 (44.2)	

Age correlated modestly with mental distress (r = 0.08, p < .000), but not with hospital admission or self-rated health. Age correlated with exposure to parental alcohol intoxication; however, the correlation was weak (r = 0.16, p < .000).

### 3.2 Multivariable analyses

**Fig. 1:**
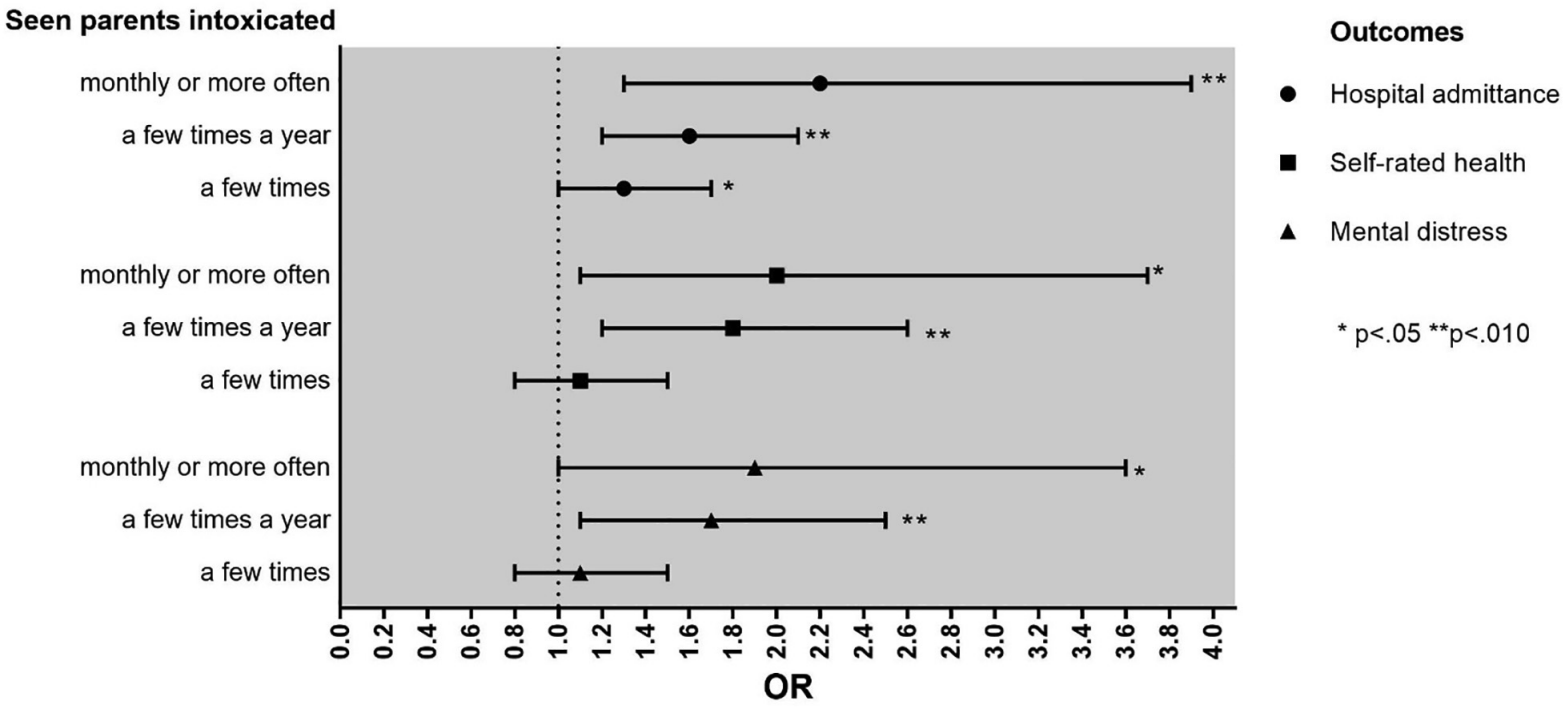
Illustration of multivariable associations between parental alcohol intoxication and adverse health outcomes among offspring. Multivariable logistic regression analyses are adjusted for gender, parental education and age. Young-HUNT1 &2, 1995–97 and 2000.

Table 3 and Fig. 1 display the results from the multivariable logistic analyses. Lifetime hospital admission was more prevalent among adolescents who had seen their parents intoxicated a few times (odds ratio 1.3; confidence interval 1.0–1.7). Further, those who had seen their parents intoxicated a few times a year also had increased odds of lifetime hospital admission (1.6;1.2–2.1), reported poorer self-rated health (1.8;1.2–2.6), and more mental distress (1.7;1.1–2.5) four years later than those who had not seen parents intoxicated. Finally, hospital admission (2.2;1.3–3.9), poor self-rated health (2.0;1.1–3.7) and mental distress (1.9;1.0–3.6) were more common among those who had seen their parents intoxicated monthly or more often than those who had not seen their parents intoxicated.

**Table 3:** Associations between parental alcohol intoxication and adverse health outcomes among offspring. Multivariable logistic regression analyses adjusting for gender, parental education and age are applied. The adolescent part of the Nord-Trøndelag Health Survey (Young-HUNT1 &2) 1995–97 and 2000.

		Self-rated poor health (n = 2042)	Mental distress (n = 2045)	Hospital admission (n = 868)
		OR (CI95%)	OR (CI95%)	OR (CI95%)
Exposure variable	Seen parents intoxicated a few times	1.1 (0.8–1.5)	1.1 (0.8–1.5)	1.3 (1.0–1.7)*
	Seen parents intoxicated a few times a year	1.8 (1.2–2.6)**	1.7 (1.1–2.5)**	1.6 (1.2–2.1)**
	Seen parents intoxicated monthly or more often	2.0 (1.1–3.7)*	1.9 (1.0–3.6)*	2.2 (1.3–3.9)**
Control variables	Gender (female reference)	0.7 (0.5–0.9)**	0.4 (0.3–0.6)***	1.2 (1.0–1.5)
	Age	1.0 (0.8–1.1)	1.4 (1.2–1.7)***	1.0 (0.9–1.1)
	Parental education, high	0.8 (0.6–1.1)	1.2 (0.9–1.6)	1.2 (1.0–1.5)

* _p<.05._

** _p<.010._

*** _p<.001._

Previous research has shown that parental alcohol use may be associated with different outcomes for female and male offspring (Haugland, 2013, Haugland et al., 2015). Therefore, we performed multivariable analyses, including an interaction term between gender and parental alcohol intoxication. However, no interactions were found, with one exception where *gender × seen parents intoxicated a few times a year* was significant (p < .05). Analyses stratified by gender revealed that girls who reported seeing their parents intoxicated a few times a year had increased odds of mental distress (2.3;1.4–3.6), but not boys (0.9;0.4–1.9).

Age was not associated with self-reported health or hospital admission. However, mental distress increased with increasing age regardless of exposure of parental alcohol intoxication, parental education, or gender. Parental education was not associated with either of the outcome variables.

## 4 Discussion

This prospective study shows that exposure to parental alcohol intoxication reported in early adolescence was associated with adverse health outcomes four years later. Exposure to parental alcohol intoxication, even just a few times a year, is associated with poor self-rated health, mental distress and having been hospitalised. For hospital admission, there was also a relationship with having seen parents intoxicated ever. There was also a tendency that these adverse outcomes increased relative to how often the adolescents had been exposed to parental alcohol intoxication.

At first sight, these findings would seem to be unremarkable in the context of the known harm caused by parental substance misuse demonstrated through previous research that found heavy parental drinking to be significantly linked with child harm measures, (Rossow, 2016, McGovern, 2018, Yap, 2017). However, this study contributes to existing knowledge by showing that adverse health effects may also be associated with more common non-dependent drinking patterns such as parental episodic alcohol intoxication. This is very important from a public health perspective, as the prevalence of such harm is probably higher than AHTO related to alcohol disorders.

Adolescents who had seen their parents intoxicated monthly or more often had doubled odds of reporting poor health in general. This finding is in line with other studies concerning adverse health consequences related to parental alcohol misuse (Christoffersen and Soothill, 2003), including poor self-perceived health (Balsa et al., 2009). We also found that seeing parents intoxicated only a few times a year almost had the same effect with up to doubled odds of poor self-rated health among these adolescents. This finding is more novel as few studies have investigated adverse health outcomes related to such prevalent but still potentially hazardous drinking patterns (McGovern, 2018).

The participants in our study had increased odds of lifetime hospital admission if they had seen their parents intoxicated by the time of early adolescence, regardless of the frequency of parental alcohol intoxication. This also corresponds to previous findings regarding alcohol-related hospital admission associated with parental substance abuse (Raitasalo, 2015). Several studies find that parental problematic alcohol use is associated with various types of injuries (Orton, 2012, Winqvist, 2007, Bijur, 1992), which may lead to hospitalisation (Winqvist, 2007). Why children of parents with problematic alcohol use more often experience injuries or are hospitalised is not explored in our study. However, studies show that parental drinking is related to a range of negative parenting behaviours such as harsh parenting, maltreatment, neglect and inattentiveness (Lang, 1999, Famularo et al., 1992, Freisthler et al., 2015, Kim, 2010, Dube, 2001) that may increase risk of health harm.

A Young-HUNT/HUNT study previously found a relationship between maternal alcohol abuse and mental distress in offspring (Rognmo, 2012). This current study shows that such associations also occur when more common drinking behaviours like parental alcohol intoxication are investigated, and that self-reported mental distress increased even at a low frequency of exposure to parental alcohol intoxication. Another prospective study based partly on the same population linking health registry data (outcomes) with HUNT survey data investigated whether different constellations of early parental risk, characterised by drinking, mental health, and education were associated with children’s subsequent diagnoses or treatment of anxiety or depression. Although they did not study parental alcohol intoxication, the study included subclinical parental drinking patterns. It concluded that although the level of each individual risk was not high, it contributed to an increase in the risk of mental health problems among children when accumulated at a family level (Lund, 2019). Raitasalo (2019) investigated the incidence of mental disorders over time among the children of parents with different levels of alcohol problems (no problems, less severe and severe alcohol problems). They found that mental disorders are associated with parental alcohol use irrespective of the severity of the drinking problem. In contrast to these studies, a recent UK study of a birth cohort (ALSPAC) could not demonstrate an association between high maternal weekly alcohol intake (over 21 units) and offspring’s conduct and depressive symptoms (Mahedy, 2017). Why parental drinking may cause mental distress among children, is not explored in the current study. However, being together with an intoxicated parent may cause emotional harm as the interaction between a drunk parent and its child deviates from the normal. Often, a child feels responsible for its intoxicated parent and experiences stress and anxiety because of this. Children tend to keep such issues hidden due to loyalty, fear, or communicative limitations and may take on a caring role towards parents and siblings (Aneta and Schier, 2012).

In summary, the more adolescents experienced parental alcohol intoxication, the higher their odds of being admitted to hospital, rating their general health as poor and experiencing mental distress. While relatively small numbers of adolescents reported the most worrying levels of parental alcohol intoxication, as noted above, there is a gradient, with adolescents reporting increasing adverse health outcomes in line with increasing parental alcohol intoxication. These impacts can be seen even with a relatively low frequency of parental alcohol intoxication.

This is a prospective study based on a large, representative population of Norwegian adolescents investigating questions on which previous research is sparse; as such it contributes to knowledge on how non-dependent drinking patterns among parents may affect the health of their offspring.

Measures are based solely on self-report and may be affected by various forms of bias (Johnson, 2014). Firstly, parents’ and adolescents’ accounts of their behaviour are subjective, and thus the same behaviour may be evaluated differently. Secondly, data may be limited by factors such as underreporting, recall bias, selective reporting and seasonal variations in lifestyle behaviour (World Health Organization, 2000). Thirdly, questions may also be considered sensitive, and the potential threat of disclosure to a third party and the aspect of social desirability may impact responses. Nonetheless, studies have shown that school environment and self-report measures of behaviour are relevant to evaluating adolescent lifestyle and health (Pisinger et al., 2016, Aas et al., 1996, Van der Vorst, 2013). Another study limitation is the inability to distinguish parental gender or whether respondents are referring to biological parents, step-parents or other caregivers when reporting on parental alcohol intoxication. We have also not included family structure, and results must be interpreted with this limitation in mind. However, around 80% of the respondents live with both parents, and studies from similar populations (YOUNG-HUNT3) found that the relationship between parental intoxication and adverse outcomes among adolescents remains strong even when separation/divorce in the family is accounted for (Haugland et al., 2015).

Confidence intervals are quite broad, which may be due to a limited number of respondents in some of the categories. Although this leaves us with less precise effect estimates for these groups, the main contribution of this study is the patterns that emerge in relation to less frequent parental alcohol intoxication and adolescent health. In these groups, the confidence intervals are narrower.

The dataset is 20 years old. However, research during this period repeatedly shows a relationship between parental alcohol use and adverse outcomes among children (Rossow, 2016, McGovern, 2018).

## 5 Implications

Over half of the adolescents in this study reported that they had seen a parent intoxicated, although only 4% reported seeing their parents alcohol intoxicated monthly or more often. If exposure to parental alcohol intoxication increases the risk of health harm in adolescence, public health measures should focus on broader alcohol use patterns among parents rather than alcohol use disorders only.

## 6 Conclusions

The adolescents who had seen their parents intoxicated had been admitted to hospital more often, reported poorer self-rated health and more mental distress. Findings indicate that adverse health outcomes among adolescents are related to relatively common drinking behaviours among parents in a general population. Findings provide valuable knowledge within the research fields of adolescent health and alcohol’s harm to others. Children have limited possibilities to protect themselves in situations where parental alcohol use causes harm. Therefore, they represent a particularly vulnerable group. The identification of factors that may affect adolescent health is essential for the development of preventive approaches to reduce adverse health outcomes in adolescence.

## Funding sources

This research did not receive any specific grant from funding agencies in the public, commercial, or not-for-profit sectors.

## Footnotes

1 As the study just completed the data collection of HUNT4, there is not an updated cohort-profile reference to cite. The number was provided to us by email from the HUNT-Study.
